# Peer Victimization and Non-Suicidal Self-Injury Among Chinese Adolescents: Moderating Roles of Teacher Emotional Support and Parent–Child Cohesion

**DOI:** 10.3390/bs16091637

**Published:** 2026-09-14

**Authors:** Ning Liu, Anyan Li, Qingxiu Xie, Mengge Yue

**Affiliations:** 1 School of Psychology, Hainan Normal University, Haikou 571158, China; 202313045116025@hainnu.edu.cn (A.L.); 202313045116027@hainnu.edu.cn (Q.X.); 202313045116010@hainnu.edu.cn (M.Y.); 2 Hainan Provincial Research Base for Psychological Development and Education of Adolescents, Haikou 571158, China; 3 Key Laboratory of Data Science and Smart Education, Ministry of Education, Hainan Normal University, Haikou 571127, China

**Keywords:** non-suicidal self-injury, peer victimization, sleep quality, parent–child cohesion, teacher emotional support, adolescents

## Abstract

Non-suicidal self-injury (NSSI) is a highly prevalent maladaptive psychological behavior among adolescents and a significant public health concern. Drawing upon ecological systems theory, this cross-sectional study of 4128 adolescents (M = 12.78, SD = 0.96; 51.6% male) aimed to investigate the underlying mechanisms and protective roles of peer victimization, sleep quality, parent–child cohesion, and teacher emotional support in relation to NSSI. Hierarchical regression analyses and PROCESS macro were used to test the proposed mediating and moderating effects. Peer victimization was significantly correlated with NSSI, and this relationship was mediated by sleep quality. In separate models, the interaction terms of peer victimization × parent–child cohesion, sleep quality × parent–child cohesion, and sleep quality × teacher emotional support were each significantly associated with NSSI. However, when both moderators were examined simultaneously, only teacher emotional support remained a significant moderator of the sleep quality–NSSI pathway. These findings highlight the complex pathways linking peer victimization to NSSI, clarify the differentiated moderating roles of family and school support, and provide empirical support for ecological systems theory and school-based interventions.

## 1. Introduction

Non-suicidal self-injury (NSSI) is defined as the direct, deliberate, and repeated destruction of one’ s own body tissue without suicidal intent (Nock, 2010). NSSI typically emerges in early adolescence, with a mean onset age of approximately 11.35 years (Thompson et al., 2026), and is particularly prevalent among middle school students aged 11 to 15. Recent research indicates that the lifetime prevalence of NSSI among Chinese adolescents is as high as 24.7% (D. Qu et al., 2023). Given that NSSI poses a serious threat to adolescents’ mental and physical health, is strongly associated with negative emotional outcomes (Hatzopoulos et al., 2022), and is linked to an elevated risk of suicide (X. Zhang et al., 2024), it has become a critical public health concern. Understanding the mechanisms underlying adolescent NSSI is therefore essential for developing effective prevention and intervention strategies.

### 1.1. Peer Victimization and Non-Suicidal Self-Injury

Peer victimization refers to an individual’s experience of being subjected to harmful behaviors by peers, which can range from spreading rumors to physical assault (Mynard & Joseph, 2000). The interpersonal model of NSSI posits that individuals who suffer from a variety of interpersonal problems, such as peer victimization, tend to use NSSI as a coping method to relieve tension and escape from emotional distress (Nock, 2010). Empirical studies in China have shown that peer victimization is significantly associated with NSSI behavior in junior high school students (H. Liu et al., 2023; M. Qu et al., 2023; Wu et al., 2024). Accordingly, the first hypothesis was proposed for this study.

**H1.**  *Peer victimization is positively associated with NSSI in adolescents.*

### 1.2. Sleep Quality as a Mediator

The context–process–outcomes model suggests that adverse environmental factors are associated with sleep quality in adolescents and that sleep problems are linked to adverse behavioral outcomes such as NSSI. Sleep quality has both objective (e.g., sleep duration and efficiency) and subjective (e.g., perceived sleep depth and restfulness) aspects (Buysse et al., 1989). According to psychological stress theory, peer victimization acts as a chronic stressor that activates the HPA axis. This activation increases cortisol levels, which disrupts circadian rhythms and ultimately alters sleep architecture (e.g., by reducing deep sleep) (Bakth et al., 2025). It is therefore reasonable to suggest that the association between peer victimization and NSSI may be mediated by sleep quality (Roeser et al., 1996).

The stress–sleep relationship theory points out that under stress, stress impairs sleep quality, and this impaired sleep in turn becomes an additional source of stress, thus creating a vicious cycle (Yan et al., 2010). Empirical studies have shown an increased likelihood of subsequent NSSI behaviors when adolescents experience sleep problems such as insomnia and frequent nightmares (Bauducco et al., 2025). Accordingly, the following hypothesis is proposed.

**H2.**  *Sleep quality mediates the association between peer victimization and NSSI in adolescents.*

### 1.3. Parent–Child Cohesion as a Moderator

Ecological systems theory identifies the family and school environments as key microsystems that influence individual development (Bronfenbrenner, 1992). Accordingly, the association between peer victimization and adolescent NSSI mediated by sleep quality may vary depending on perceived parent–child cohesion and perceived teacher emotional support.

Parent–child cohesion is defined as the emotional bonding and support among family members, reflecting closeness and resilience within the family unit (Olson, 2000). According to the stress-buffering hypothesis (SBH), support from others (e.g., perceived high parent–child cohesion) can buffer the association between adverse experiences (e.g., peer victimization) and individuals’ physical and mental development (S. Cohen & Wills, 1985). Studies have shown that adolescents who perceive high parent–child cohesion show decreased HPA-axis hyperactivated inflammation, resulting in improved quality of sleep and thus a reduction in self-injurious impulses (Barnert et al., 2015). Accordingly, Hypothesis 3 is proposed for this study:

**H3.**  *Parent–child cohesion moderates the mediated association between peer victimization and NSSI through sleep quality, including both the indirect path (through sleep quality) and the direct path.*

### 1.4. Teacher Emotional Support as a Moderator

Teacher emotional support is a form of social support that effectively buffers the negative association between peer victimization and adverse outcomes (Galindo-Domínguez & Losada, 2026). It refers to the care, respect, and understanding that students perceive from their teachers (Asher et al., 1998). When adolescents perceive high teacher emotional support in the context of peer victimization, they are less likely to develop internalizing problems, including NSSI (Y. Liu et al., 2024; Guo et al., 2022).

Studies have shown that perceived positive school climate (e.g., teacher support) is associated with better sleep quality, which in turn is associated with a lower likelihood of suicidal behavior (Xu et al., 2025). Accordingly, the following hypothesis is proposed:

**H4.**  *Teacher emotional support moderates the mediated association between peer victimization and NSSI through sleep quality, including both the indirect path (through sleep quality) and the direct path.*

### 1.5. The Double-Moderating Effect of Parent–Child Cohesion and Teacher Emotional Support

The protective–protective model suggests that there may be either an augmentative or antagonistic pattern of interaction between two protective factors when they act together on adolescent development (J. Cohen et al., 2003). For example, when individuals perceive high levels of parent–child cohesion, teacher emotional support might not make a significant additional contribution to their healthy physical and mental development. Research exploring the interactive roles of school and family factors in relation to suicidal ideation among college student victims of bullying has found teacher emotional support to be a protective factor and family support to be a significant moderator (J. W. Zhang et al., 2024). However, other studies support an alternative model. For example, the findings of Chen et al. (2017) suggest that parental support is positively associated with friendship quality for individuals with low school connectedness but not for those with high school connectedness. Given the lack of research on the potential synergistic effects of parent–child cohesion and teacher emotional support, this study examines the moderating roles of both factors in the relationship between peer victimization and NSSI without specifying a priori assumptions about their interaction patterns.

In summary, based on the abovementioned theoretical considerations and empirical findings, this study among middle school students in China examines the associations between peer victimization and adolescents’ NSSI behaviors, with consideration of the roles of sleep quality, parent–child cohesion, and teacher emotional support. This study aims to provide empirical evidence that can inform targeted prevention and intervention efforts for NSSI in adolescents.

## 2. Method

### 2.1. Study Design and Participants

This was a cross-sectional study conducted among secondary school students in China. The provinces of Hainan, Sichuan, and Henan were purposively selected to represent the southern, southwestern, and central regions of China, respectively, based on geographic distribution and regional economic development levels. Within each province, one prefecture-level city was selected, and a total of nine secondary schools were recruited across the three cities through convenience sampling, based on accessibility and school administrators’ willingness to participate.

The inclusion criteria for participants were: (1) currently enrolled students in grades 7 and 8 at the selected schools (grade 9 students were not included due to the academic pressure associated with the high school entrance examination); (2) aged between 11 and 14 years; and (3) willingness to participate, with provision of written informed consent from both students and their guardians. The exclusion criteria were: (1) severe physical or mental illness that prevented completion of the questionnaire; (2) cognitive or reading difficulties that interfered with understanding the survey items; (3) absence from school on the day of survey administration; and (4) enrollment in grade 9.

A total of over 4000 questionnaires were distributed. After excluding invalid responses, 4128 valid questionnaires were finally collected, yielding an effective response rate of 98%. Among the valid sample, 3946 participants reported their gender, including 2036 male students (51.6% of those who reported gender) and 1910 female students (48.4% of those who reported gender). The remaining 182 participants (4.4% of the total sample) did not report their gender. The mean age of the sample was 12.78 ± 0.956 years.

### 2.2. Instruments and Measures

Adolescent Non-suicidal Self-injury Assessment Questionnaire (ANSAQ). The questionnaire, which was revised by Wan et al. (2018) for the Chinese cultural context, was adopted in this study. The original ANSAQ is composed of two subscales: the Self-Injurious Behavior Subscale (12 items) and the Functional Subscale (19 items). To reduce participant fatigue, only the Self-Injurious Behavior Subscale was retained, and participants were asked to rate the frequency of 12 self-injurious behaviors (e.g., “deliberately pinching oneself”). A 5-point Likert scale was employed, ranging from 0 (never) to 4 (always), with higher total scores indicating more frequent non-suicidal self-injury. In this study, Cronbach’s α for this subscale was 0.924.

Multidimensional Peer-Victimization Scale (MPVS). The Chinese version of the Multidimensional Peer Victimization Scale, developed by W. Zhang et al. (2009), was adopted in this study. This scale consists of 11 items (e.g., “Other students threatened to beat me this semester”), which are rated on a 4-point Likert scale ranging from 1 (never occurred) to 4 (frequently occurred). It was used to assess students’ experiences of peer victimization over the past six months, with higher total scores indicating more severe peer victimization. In this study, Cronbach’s α for this scale was 0.877.

Pittsburgh Sleep Quality Index (PSQI). This scale was developed by Buysse et al. (1989) and was used to assess the sleep quality of the participants over the previous month (e.g., “During the past month, how often have you had trouble sleeping because you cannot get to sleep within 30 min?”). The 18 self-rated items are scored on a 4-point scale ranging from 0 to 3, yielding a global score ranging from 0 to 21, with higher scores indicating poorer sleep quality. Chinese researchers have tested the PSQI and confirmed its validity and reliability for Chinese samples (X. Liu et al., 1996). In this study, Cronbach’s α for this scale was 0.651.

Parent–Child Cohesion Scale. The Chinese revised version of the Parent–Child Cohesion Scale, which has been found to meet psychometric requirements (W. Zhang & Wang, 2005), was used to measure parent–child cohesion. The scale consists of 10 items, of which items 3, 4, 8, 9, 13, 14, 18, and 19 are reverse-scored (e.g., “I feel closer to other people than to my parents”). A 5-point Likert scale was employed, ranging from 1 (never) to 5 (always). During the calculation, the average score of the father–son affinity and mother–son affinity was taken as the total score of parent–child affinity. The higher the score, the better the quality of the parent–child relationship. In this study, Cronbach’s α for this scale was 0.887.

Middle School Students’ Questionnaire on Perceiving Emotional Support from Teachers. This questionnaire was compiled by Gao et al. (2017) and was used to measure teacher emotional support. The questionnaire consists of 18 items (e.g., “My teacher understands my difficulties”), rated on a 5-point Likert scale ranging from 1 (strongly disagree) to 5 (strongly agree). Higher total scores indicate higher levels of perceived teacher emotional support among students. In this study, Cronbach’s α for this scale was 0.970.

### 2.3. Procedure

After obtaining consent from school administrators and the students themselves, graduate students majoring in psychology served as examiners. Each assessment was conducted outside of the start-of-semester and exam periods, in class-wide sessions. The questionnaire took approximately 30 min to complete and was collected on-site immediately afterward. Prior to the test, the examiners provided detailed instructions and precautions, emphasizing that there were no right or wrong answers and encouraging students to respond honestly based on their actual circumstances. All participants were assured that the collected data would be kept strictly confidential and used solely for research purposes.

Ethical approval for this study was obtained from the Ethics Committee of University (Approval No.: 202401026). All participants and their legal guardians were fully informed of the purpose and procedures of the study. Written informed consent was obtained from participants and their guardians prior to their participation.

### 2.4. Bias

This study employed both measurement control and statistical control during the administration process to mitigate serious common method bias. Measurement controls included: (1) anonymous responding; (2) the use of different rating scales for different variables; and (3) reverse-scoring for some items. Statistical control: Prior to formal data analysis, Harman’s single-factor test was conducted on the raw data to examine common method bias. The results show that the first common factor accounted for 21.7% of the variance, which is below the 40% threshold. This provides preliminary evidence that common method bias may not be a substantial concern, although the test cannot fully rule out its presence.

### 2.5. Statistical Methods

Prior to the main analyses, data screening was performed. Cases with incomplete responses on key study variables (peer victimization, NSSI, parent–child cohesion, teacher emotional support, and sleep quality) were excluded from the analyses. No missing data imputation methods were applied, as the proportion of missing data was minimal after exclusion of invalid cases. Statistical analyses were conducted using SPSS 29.0 software, and mediation and moderation effect tests were performed using the PROCESS macro for SPSS developed by Hayes.

In the first step, Pearson correlation analysis was conducted using SPSS 29.0 to examine the correlations among all variables.

In the second step, Model 4 of the PROCESS macro was used to test the simple mediation effect.

In the third step, hierarchical regression analysis was performed using SPSS to test the moderating effect. Specifically, Specifically, Model 59 was used to test the moderating role of parent–child cohesion, estimating moderation on all three pathways (peer victimization → sleep quality, sleep quality → NSSI, and peer victimization → NSSI), whereas Model 58 was used to test the moderating role of teacher emotional support, estimating moderation on the first and second stages (peer victimization → sleep quality and sleep quality → NSSI) only.

In the fourth step, Model 65 of the PROCESS macro was further used to verify the hypothesized dual moderation model. Model 65 was selected because it allows for the inclusion of two moderators simultaneously and estimates their interactive effects on the first stage, second stage, and direct path of the mediation model, thereby testing whether parent–child cohesion and teacher emotional support jointly moderate the indirect association between peer victimization and NSSI.

## 3. Results

### 3.1. Correlations Among the Variables

As shown in Table 1, peer victimization was positively correlated with the frequency of self-injurious behavior (*r* = 0.292, *p* < 0.001) and with poor sleep quality (*r* = 0.288, *p* < 0.001). Perceived parent–child cohesion (*r* = −0.094, *p* < 0.001) and teacher emotional support (*r* = −0.226, *p* < 0.001) were negatively associated with peer victimization.

### 3.2. Mediation Effect Test

This study, based on Model 4 of the PROCESS macro, examined the mediating role of sleep quality in the relationship between peer victimization and non-suicidal self-injury (NSSI), with gender, grade level, ethnicity, and family type included as control variables in the model. The bias-corrected percentile bootstrap method with 5000 resamples was employed to estimate the confidence intervals of the indirect effects.

As shown in Table 2, there was a direct effect of peer victimization on NSSI [0.191, 0.250] and an indirect effect through sleep quality [0.062, 0.091]. H1 is verified. This indicates that sleep quality mediated the relationship between peer victimization and NSSI, thus supporting H2. The direct and indirect effects accounted for 74% and 26% of the total effect, respectively.

### 3.3. Test of the Moderating Role of Parent–Child Cohesion

Hierarchical regression analyses were conducted in SPSS to examine the moderating role of parent–child cohesion in the main effect.

The results of the multiple linear regression analysis are shown in Table 3. Peer victimization was positively associated with NSSI in Model 2 (*B* = 0.327, *t* = 18.726, *p* < 0.001), and the interaction term between peer victimization and parent–child cohesion was significant for NSSI in Model 3 (*B* = −0. 056, *t* = −2.542, *p* < 0.05). These findings demonstrate the moderation of parent–child cohesion in the main effect, thus supporting H3a. Parent–child cohesion moderated the relationship between peer victimization and NSSI, such that the positive association between peer victimization and NSSI was weaker in students who perceived higher parent–child cohesion.

To test the moderated mediation models, Model 59 of the PROCESS macro was used to test whether parent–child cohesion moderated the stages of the indirect relationship between peer victimization and NSSI. The results are shown in Table 4. The interaction term of peer victimization and parent–child cohesion was not significantly associated with sleep quality (*β* = 0.014, *t* = 0.632, *p* > 0.05); therefore, H3b is not supported. However, the interaction term of peer victimization and parent–child cohesion (*β* = −0.046, *t* = −2.099, *p* <0.05) and the interaction term of sleep quality and parent–child cohesion (*β* = −0.042, *t* = −2.277, *p* < 0.05) were significantly associated with NSSI, supporting H3c.

### 3.4. Test of the Moderating Role of Teacher Emotional Support

Hierarchical regression analyses were also conducted through SPSS to examine the moderating role of teacher emotional support in the main effect.

The results of the multiple linear regression analysis are shown in Table 5. Peer victimization was positively associated with NSSI in Model 2 (*B* = 0.305, *t* = 17.354, *p* < 0.001) but the interaction term between peer victimization and teacher emotional support was not significantly associated with NSSI in Model 3 (*B* = −0.015, *t* = −0.236, *p* > 0.05). This finding indicates that teacher emotional support did not play a moderating role in the direct pathway between peer victimization and NSSI; therefore, H4a is not supported.

To test the moderated mediation models, Model 58 of the PROCESS macro was used to test whether teacher emotional support moderated the stages of the indirect relationship between peer victimization and NSSI. The results are shown in Table 6. The interaction term of peer victimization and teacher emotional support was not significantly associated with sleep quality (*β* = 0.034, *t* = 1.922, *p* > 0.05); therefore, H4b is not supported. The interaction term between sleep quality and teacher emotional support was significantly associated with NSSI (*β* = −0.069, *t* = −4.784, *p* < 0.001), supporting H4c.

### 3.5. Test of the Double-Moderated Mediation

Further analyses were conducted to explore whether parent–child cohesion and teacher emotional support would simultaneously moderate the mediating effect in a single model. Model 65 in PROCESS was used to validate the hypothesized double-moderation model.

The results of these analyses are shown in Table 7. With both parent–child cohesion and teacher emotional support in the model, peer victimization was positively associated with sleep quality (*β* = 0.255, *t* = 4.049, *p* < 0.001) and with NSSI (*β* = 0.346, *t* = 5.518, *p* < 0.001), and sleep quality was positively associated with NSSI (*β* = 0.535, *t* = 8.184, *p* < 0.001). The model estimation results further validate the mediating role of sleep quality. Parent–child cohesion played no moderating role in the direct effect or in the first or second segments of the mediated pathway; teacher emotional support moderated the second segment of the mediated pathway (*β* = −0.063, *t* = −4.044, *p* < 0.001).

## 4. Discussion

### 4.1. Relationship Between Peer Victimization and NSSI

The present findings provide further support for the association between adolescents’ exposure to peer victimization and NSSI behaviors, which is consistent with the findings of existing studies (Wu et al., 2024; Q. Wang et al., 2020). According to the social connection and need to belong theory, peer victimization undermines an individual’s sense of social belonging and leads to self-punishing behaviors (Simundic et al., 2024). For example, individuals who have been chronically victimized by their peers may internalize external negative evaluations (e.g., “I deserve to be hurt,” “I don’ t deserve to be loved”), and may in turn use NSSI to punish themselves as a way to alleviate internalized shame and self-loathing (Nock & Favazza, 2009).

### 4.2. Mediating Role of Sleep Quality

The finding that sleep quality mediated the relationship between peer victimization and NSSI is consistent with previous research (Tampke et al., 2019). Within the frameworks of the stress-response model (Folkman & Lazarus, 1988; Lazarus, 1993) and the experiential avoidance model (Chapman et al., 2006), prior studies have proposed that peer victimization, as a chronic social stressor (Agnew, 2001), may increase cognitive and emotional arousal at bedtime, thereby interfering with restorative sleep. Poor sleep quality, in turn, has been associated with impaired emotion regulation and reduced cognitive flexibility in previous research (Pace-Schott et al., 2017), which may increase susceptibility to maladaptive coping behaviors such as NSSI. Additionally, experimental evidence has suggested that sleep deprivation may affect pain perception and reward processing, potentially contributing to the reinforcement of NSSI (Chang et al., 2022; Nock & Favazza, 2009). Importantly, however, the present study did not directly assess any of these neurobiological or cognitive mechanisms. The above interpretations are therefore offered as theoretically grounded hypotheses derived from prior literature, not as conclusions demonstrated by our data. Future research incorporating physiological and neurocognitive measures is needed to rigorously test these proposed pathways.

### 4.3. Moderating Effect of Parent–Child Cohesion and Teacher Emotional Support

The findings on the moderating effects of parent–child cohesion and teacher emotional support are inconsistent with the hypotheses. The moderating role of parent–child cohesion on the direct pathway can be explained as follows. According to social signaling theory (Slavich, 2020), perceived high parent–child cohesion may undermine adolescents’ interpretations of peer victimization as a social threat, as family support promotes positive reappraisal (e.g., “Being bullied does not mean that I am worthless”) and reduces self-critical thinking that drives self-injurious behavior (Nolen-Hoeksema, 2012). Furthermore, a sense of family belonging can satisfy the basic psychological needs of students who are ostracized at school, thereby reducing their motivation for NSSI (Yang et al., 2025). Thus, parent–child cohesion moderates the direct pathway between peer victimization and NSSI.

The buffering role of parent–child cohesion in the association between sleep quality and NSSI is consistent with attachment theory (Bowlby, 1980). From this perspective, high parent–child cohesion may provide a secure base that facilitates adaptive coping strategies (e.g., seeking emotional support) when adolescents experience sleep-related emotional distress. In contrast, the association between peer victimization and sleep quality may involve physiological stress pathways, as suggested by prior research (McEwen, 2007). However, because the present study did not directly assess HPA-axis activity or cortisol levels, this interpretation remains speculative and should be viewed as a hypothesis derived from the literature rather than a conclusion drawn from our data. This may offer a tentative explanation for why parent–child cohesion did not buffer the association between peer victimization and sleep quality in our study. Given the cross-sectional design and the absence of biological measures, this interpretation requires further empirical testing.

Second, teacher emotional support moderated the indirect path from sleep quality to NSSI. This finding is consistent with the social support buffering hypothesis (S. Cohen & Wills, 1985) and emotion regulation theory, which suggest that poor sleep quality may compromise emotion regulation and thus increase NSSI risk (Bauducco et al., 2025; S. Li et al., 2026), and that teacher support may help adolescents manage sleep-related emotional distress, potentially reducing the likelihood of NSSI. Consistent with this pattern, Xu et al. (2025) found that perceived positive school climate (e.g., teacher support) was associated with better sleep quality, and better sleep quality was associated with a lower likelihood of NSSI.

The absence of a moderating role for teacher emotional support in the relationship between peer victimization and sleep quality may reflect the relatively strong and direct association between peer victimization and sleep quality, which could make it difficult for external support resources such as teacher emotional support to function as a significant buffer. This finding is consistent with previous empirical research. For example, Yin et al. (2025) found a strong association between peer victimization and NSSI among adolescents, as well as a limited moderating role of external support.

The finding that teacher emotional support did not moderate the peer victimization–sleep quality relationship is consistent with the stress–sleep model, which posits that external stressors may impair sleep quality through physiological and psychological stress responses (Cook et al., 2026). Prior research suggests that the association between peer victimization and sleep quality may involve stress-related physiological and psychological processes. However, because the present study did not directly assess these underlying mechanisms, this interpretation remains speculative. It is possible that teacher emotional support, which operates primarily at the psychosocial level, may have limited capacity to buffer these stress-related pathways.

Third, the double-moderating role of parent–child cohesion and teacher emotional support was examined. Neither the direct pathway nor the two indirect paths were moderated by parent–child cohesion, whereas teacher emotional support significantly moderated the sleep quality → NSSI path. These findings are broadly consistent with the protective factor–protective factor model (Fergus & Zimmerman, 2005), which suggests that different protective resources may operate through distinct mechanisms. Previous research has suggested that family support (e.g., parent–child cohesion) may be more effective in mitigating long-term risks associated with peer victimization through emotional security, rather than intervening in immediate stress responses such as sleep problems (M. T. Wang & Eccles, 2012). In contrast, teacher emotional support may act more directly on daily emotion regulation and stress management, potentially reducing the likelihood of maladaptive responses following poor sleep quality (P. Li et al., 2026). This pattern may reflect the timing of teacher support at a later stage of stress transmission, where emotional support may be more readily perceived as effective (S. Cohen & Wills, 1985). Thus, the path-specificity observed in this study may reflect the distinct roles of different microsystem resources (e.g., family and school), as suggested by ecological systems theory (Bronfenbrenner, 1979), wherein teacher emotional support may play a more prominent moderating role in the sleep quality–NSSI pathway.

## 5. Research Significance and Prospects

The findings of this study have both theoretical and practical implications. At the theoretical level, first, by introducing sleep quality as a mediating variable, this study helps to elucidate the indirect statistical association through which peer victimization may be related to NSSI, offering an integrated biopsychosocial perspective for understanding this relationship. Second, the findings support the applicability of the family/school co-protection model, extend the explanatory power of ecological systems theory (Bronfenbrenner, 1979) in the field of adolescent mental health and illustrate how resources from different systems may interact to buffer risk. Third, this study contributes to the dynamic modeling of developmental psychopathology. Whereas traditional mediation models focus primarily on unidirectional risk transmission, the introduction of a double-moderation mechanism allows for a more dynamic representation of how risk and protective factors interact. This hierarchical moderation framework may provide empirical support for dynamic models in developmental psychopathology. At the practical level, first, the significant mediating role of sleep quality suggests that clinical interventions should prioritize adolescent sleep hygiene (e.g., using cognitive behavioral therapy to address insomnia). Second, the differential moderating roles of parent–child cohesion and teacher support provide a basis for selecting between family-first or school-led intervention models.

Nonetheless, this study has some limitations that should be addressed in subsequent studies. First, due to the cross-sectional design, the findings reflect statistical associations among the variables at a single time point and do not permit causal inferences. Future research could adopt longitudinal tracking designs or cross-lagged panel analyses to examine the directional nature of these relationships. Second, the sample was recruited from three provinces (Hainan, Sichuan, and Henan) and nine secondary schools using convenience sampling. Although the selected provinces cover different geographic regions of China, the schools and participants were not randomly selected, which may limit the generalizability of the findings to the broader adolescent population. Future studies with larger, more representative, and randomly sampled populations are needed to enhance the external validity of these findings. Third, Cronbach’s α for the sleep quality scale used in this study was 0.651, which is slightly below the conventional threshold of 0.70. Although this coefficient is within the acceptable range for social science research (Taber, 2018), it may reflect the challenges of applying cross-cultural measurement tools. For instance, sleep quality dimensions derived from Western scales may not fully align with the sleep schedules and cultural practices of Chinese adolescents, such as the impact of group living arrangements on sleep (Tsai et al., 2005). More importantly, as sleep quality was assessed as the mediating variable in this study, the relatively lower reliability may have attenuated the estimated indirect associations, meaning that the true mediating effect of sleep quality could be larger than observed (MacKinnon, 2008). Future studies should prioritize the use of locally validated sleep assessment instruments with higher reliability to obtain more accurate estimates of the mediation effects.

## 6. Conclusions

This cross-sectional study of 4128 middle school students examined the associations among peer victimization, sleep quality, parent–child cohesion, teacher emotional support, and non-suicidal self-injury (NSSI). The results indicate significant correlations among all variables. Mediation analysis revealed that sleep quality significantly mediated the indirect association between peer victimization and NSSI. Moderation analysis further demonstrated that, in separate models, the interaction terms of peer victimization × parent–child cohesion, sleep quality × parent–child cohesion, and sleep quality × teacher emotional support were all significantly associated with NSSI. However, when both moderators were examined simultaneously, only teacher emotional support remained a significant moderator of the sleep quality–NSSI pathway, whereas parent–child cohesion did not show a significant moderating effect. These findings extend the theoretical understanding of the pathways underlying the relationship between peer victimization and adolescent NSSI while also providing empirical evidence for early identification and tiered intervention approaches. Given the cross-sectional design, causal relationships cannot be inferred. Future research should employ longitudinal or intervention designs to examine the effectiveness of multidimensional synergistic intervention strategies, such as reducing victimization exposure, optimizing sleep conditions, and strengthening parent–child and teacher–student relationships, in relation to NSSI outcomes among adolescents.

## Figures and Tables

**Table 1:** Correlations among the study variables.

Variable	M	SD	1	2	3	4	5
1. NSSI	0.179	0.444	1				
2. Peer victimization	1.236	0.390	0.292 **	1			
3. Sleep quality	0.996	0.459	0.346 **	0.288 **	1		
4. Parent–child cohesion	2.814	0.747	−0.101 **	−0.094 **	−0.167 **	1	
5. Teacher emotional support	3.626	0.939	−0.188 **	−0.226 **	−0.276 **	0.338 **	1

*Notes*. NSSI = non-suicidal self-injury. ** *p* < 0.01.

**Table 2:** Total, direct, and indirect effect of peer victimization on NSSI via sleep quality.

	Effect	Boot SE	LLCI	ULCI	Relative Mediation Effect
Total effect	0.296	0.0152	0.266	0.326	
Direct effect	0.220	0.0151	0.191	0.250	74%
Indirect effect	0.076	0.0076	0.062	0.091	26%

*Notes*. NSSI = non-suicidal self-injury.

**Table 3:** Parent–child cohesion as moderator: Main effect test results.

Predictive Variable	NSSI					
	Model 1		Model 2		Model 3	
	B	t	B	t	B	t
Constant term	0.138	1.647	−0.142	−1.586	−0.13	−1.457
Gender	0.073	5.613 ***	0.078	6.252 ***	0.079	6.309 ***
Family type	0.016	1.781	0.013	1.535	0.013	1.456
Father’s education	−0.022	−1.866	−0.015	−1.341	−0.015	−1.390
Mother’s education	−0.013	−1.136	−0.012	−1.063	−0.012	−1.075
Ethnicity	0.082	3.625 ***	0.051	2.338 *	0.051	2.357 *
Grade	−0.001	−0.05	−0.028	−2.185 *	−0.028	−2.170 *
Boarding status	0.016	0.976	0.030	1.902	0.029	1.846
Class cadre status	−0.030	−2.085 *	−0.023	−1.635	−0.022	−1.612
Only child status	−0.018	−0.891	−0.006	−0.286	−0.006	−0.324
Left-behind Status	−0.009	−0.486	−0.011	−0.655	−0.013	−0.748
Peer victimization			0.327	18.726 ***	0.323	18.408 ***
Parent–child cohesion			−0.047	−5.142 ***	−0.047	−5.196 ***
Parent–child cohesion × peer victimization					−0.056	−2.542 *
R^2^	0.018		0.110		0.111	
F	7.295 ***		40.197 ***		37.653 ***	

*Notes*. NSSI = non-suicidal self-injury. *** *p* < 0.001; * *p* < 0.05.

**Table 4:** The moderating effect of parent–child cohesion on the mediation model.

Outcome Variables	Predictive Variables	β	SE	t	LLCI	ULCI
Sleep quality	Peer victimization	0.292	0.017	16.630 ***	0.258	0.327
	Parent–child cohesion	−0.101	0.009	−11.076 ***	−0.119	−0.083
	Peer victimization × parent–child cohesion	0.014	0.022	0.632	−0.029	0.057
NSSI	Peer victimization	0.2432	0.018	13.934 ***	0.209	0.2774
	Sleep quality	0.272	0.015	17.716 ***	0.242	0.302
	Parent–child cohesion	−0.022	0.009	−2.458 *	−0.040	−0.005
	Peer victimization × parent–child cohesion	−0.046	0.022	−2.099 *	−0.090	−0.003
	Sleep quality × parent–child cohesion	−0.042	0.019	−2.277 *	−0.080	−0.006

*Notes*. NSSI = non-suicidal self-injury. *** *p* < 0.001; * *p* < 0.05.

**Table 5:** Teacher emotional support as moderator: main effect test results.

Predictive Variable	NSSI					
	Model 1		Model 2		Model 3	
	B	t	B	t	B	t
Constant term	0.144	1.745	−0.032	−0.361	−0.03	−0.337
Gender	0.07	5.409 ***	0.078	6.297 ***	0.078	6.299 ***
Family type	0.017	1.911	0.019	2.218 *	0.019	2.221 *
Father’s education	−0.021	−1.808	−0.011	−1.009	−0.03	−1.012
Mother’s education	−0.013	−1.162	−0.006	−0.557	0.078	−0.552
Ethnicity	0.085	3.775 ***	0.04	1.858	0.019	1.864
Grade	−0.003	−0.237	−0.028	−2.242 *	−0.011	−2.242 *
Boarding status	0.016	0.995	0.031	1.979 *	−0.006	1.968 *
Class cadre status	−0.031	−2.156 *	−0.03	−2.156 *	0.04	−2.159 *
Only child status	−0.018	−0.905	−0.005	−0.271	−0.028	−0.277
Left-behind Status	−0.01	−0.573	−0.015	−0.922	0.031	−0.917
Peer victimization			0.305	17.354 ***	−0.03	15.982 ***
Teacher emotional support			−0.061	−8.375 ***	−0.005	−8.377 ***
Teacher emotional support × Peer victimization					−0.015	−0.236
R2	0.018		0.120		0.120	
F	7.280 ***		228.913 ***		0.056	

*Notes*. NSSI = non-suicidal self-injury. *** *p* < 0.001; * *p* < 0.05.

**Table 6:** The moderating effect of teacher emotional support on the mediation model.

Outcome Variables	Predictive Variables	β	SE	t	LLCI	ULCI
Sleep quality	Peer victimization	0.310	0.018	17.700 ***	0.275	0.344
	Teacher emotional support	−0.103	0.007	−14.232 ***	−0.117	−0.089
	Peer victimization × teacher emotional support	0.034	0.017	1.922	−0.001	0.068
NSSI	Peer victimization	0.231	0.017	13.275 ***	0.197	0.265
	Sleep quality	0.254	0.015	16.506 ***	0.224	0.285
	Teacher emotional support	−0.037	0.007	−5.183 ***	−0.051	−0.023
	Sleep quality × teacher emotional support	−0.069	0.014	−4.784 ***	−0.098	−0.041

*Notes*. NSSI = non-suicidal self-injury. *** *p* < 0.001.

**Table 7:** Double-moderation test results.

Outcome Variable	Predictive Variable	β	SE	t	LLCI	ULCI
Sleep quality	Peer victimization	0.255	0.063	4.049 ***	0.132	0.378
	Parent–child cohesion	−0.119	0.029	−4.151 ***	−0.175	−0.063
	Peer victimization × parent–child cohesion	0.015	0.022	0.658	−0.029	0.058
NSSI	Peer victimization	0.346	0.063	5.518 ***	0.223	0.470
	Sleep quality	0.535	0.065	8.184 ***	0.406	0.663
	Parent–child cohesion	0.061	0.030	1.999 *	0.001	0.120
	Peer victimization × parent–child cohesion	−0.042	0.022	−1.892	−0.085	0.002
	Sleep quality × parent–child cohesion	−0.019	0.020	−0.960	−0.058	0.020
	Teacher emotional support	−0.027	0.017	1.601	−0.006	0.059
	Sleep quality × teacher emotional support	−0.063	0.015	−4.044 ***	−0.093	−0.032

*Notes*. NSSI = non-suicidal self-injury. *** *p* < 0.001; * *p* < 0.05.

## Data Availability

The data that support the findings of this study are available on request from the corresponding author. The data are not publicly available due to privacy or ethical restrictions.
